# Degree of Functionality and Perception of Health-Related Quality of Life in People with Moderate Stroke: Differences between Ischemic and Hemorrhagic Typology

**DOI:** 10.1155/2019/3405696

**Published:** 2019-04-28

**Authors:** Iratxe Unibaso-Markaida, Ioseba Iraurgi, Nuria Ortiz-Marqués, Silvia Martínez-Rodríguez

**Affiliations:** Psychology Department, University of Deusto, Bilbao, 48007 Basque Country, Spain

## Abstract

**Objectives:**

The objectives of this pilot study were to analyze the functional differences and the differences regarding the perception of health-related quality of life between people affected by ischemic and hemorrhagic stroke, respectively, and between these and their normative groups.

**Methods:**

A pre-post design study was conducted with 30 patients aged 65 ± 15 during eight weeks. It assessed disability, mobility, and health-related quality of life. Exact nonparametric tests were used to compare both types of stroke, and *t*-tests and effect size estimates were employed to compare the stroke group and the normative group.

**Results:**

At baseline, there were differences in disability (“getting along” domain), where a poorer result was obtained by the hemorrhagic stroke group, and in the “vitality” and “mental health” domains of the health-related quality of life test, where the ischemic group obtained poorer results. Both groups made significant progress in their health assessments and functionality after eight weeks, and no significant differences were found between them at that time. The scores obtained in both groups differed statistically from the normative values, both at baseline and at posttest.

**Conclusions:**

Regardless of the stroke type, divergent results were only found in two domains, “vitality” and “mental health.” There was an improvement over time, but the scores obtained were still lower than those observed in the normative group, which indicated that the participants' health was highly compromised. This study provides more information for faster rehabilitation after stroke; even so, more studies are needed.

## 1. Introduction

Stroke is “a focal (or sometimes general) neurological impairment of sudden onset, and lasting more than 24 hours (or leading to death), and of presumed vascular origin” [1]. A total of 87% of strokes are ischemic, while 3% are hemorrhagic, and the remaining 10% are other forms of stroke; hemorrhagic strokes have greater mortality [2]. There are 610,000 new cases and 185,000 recurrent cases every year in the United States [2]. In Spain, it is the first cause of death in women and the second in men [3]; in Europe, there are one million new cases each year, and it is the first cause of disability worldwide [4, 5].

A total of 86% of people who have suffered a stroke have some type of disability, mostly mobility problems, followed by 39% who have communication problems and 34% who suffer from learning problems [6]. The American Heart Association has classified the disability from stroke into six domains: motor, sensory, language, visual, cognitive, and affective [7]. In addition to the domains, disability from stroke has also been classified according to levels [8].

An individual's quality of life is worse after a stroke, and in most cases they need help in their day-to-day living, which in turn affects the quality of life of family members [9]. Strokes have both physical and mental effects. In addition, there is a perception of low quality of life due to depression symptoms, low social interaction, and cognitive and functional problems following a stroke [10].

Little evidence that compares the consequences of hemorrhagic and ischemic strokes has been obtained to date. The existing studies have focused on searching for biomarkers that differentiate both typologies, on risk factors, and even on the risk of mortality that each type presents [11–13]. Taking into account the number of strokes that occur each year, more than a million in Europe, and the severity of the sequelae that leaves, it is the first cause of disability around the world [4, 5]; the objective of this study, therefore, was to compare the possible differences, in terms of functionality (disability and mobility) and the perception of health-related quality of life for each stroke type, as well as to compare these data with the normative group data set.

## 2. Methods

### 2.1. Participants

Thirty people participated in the study, of whom 20 were male and 10 were female. All of them were patients at the Hospital specializing in this type of conditions in the Biscay (Basque Country, Spain) public network. The mean age of the participants was *M* = 65 (SD = 15). Whereas 21 of the patients had suffered an ischemic stroke (15 patients suffered ischemic stroke on the right hemisphere and six patients on the left), 9 of them had suffered a hemorrhagic stroke (8 of them in the right hemisphere) (see Table 1, sociodemographic characteristics of the sample). The inclusion criteria were evaluated by the rehabilitation doctors from the participating hospital and included being of legal age (+18), being diagnosed with moderate stroke using the Oxfordshire Community Stroke Project (OCSP) classification, having a score of 60-90 on the Barthel Index and a cut-off point of 23 or higher on the Mini-Mental State Examination (MMSE), having suffered a stroke at least a month before but not longer than a year ago, having attended rehabilitation sessions at a specific hospital within the Bizkaia healthcare network, and having dominant hand that must preserve dexterity to carry out evaluation tasks. Excluded patients were those who were in an unstable clinical situation or had complications that required active medical treatment after the stroke, those who were hemodynamically unstable or had active infections, people with comprehension problems that prevented them from following verbal orders, people with dementia or cognitive impairment, and people with a nonstabilized or noncontrolled psychiatric disorder.

### 2.2. Ethical Standards

The purpose of the study was explained to all participants, who gave written consent and understood that their participation was voluntary. The study was approved by the Ethics Committee at the University of Deusto and the Ethics Committee of the Basque public health network and conforms to the ethical guidelines of the 1975 Helsinki Declaration.

### 2.3. Design and Procedure

**Figure 1:**
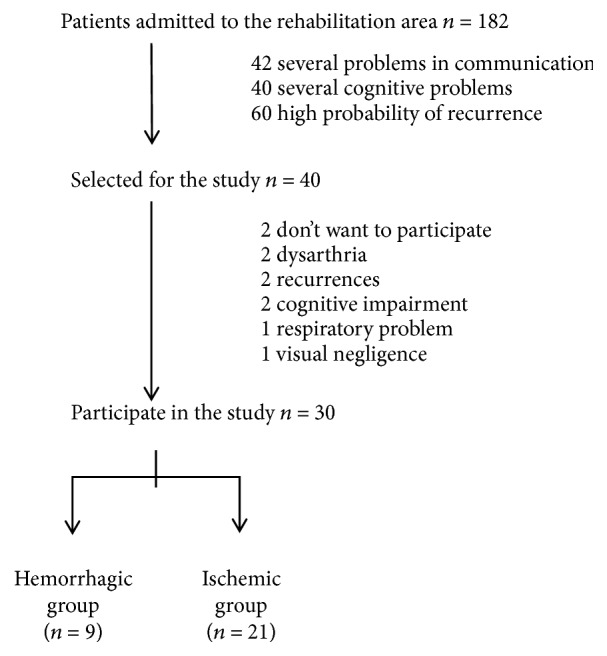
Sample selection.

The study employed a pre-post design. A total of 30 patients who were undergoing rehabilitation out of a total of 182 people between June 2016 and February 2017 participated in the study (see Figure 1, selection flow chart). They were asked for informed consent to participate in the study and were assigned a numerical code to ensure that the anonymity of the data was maintained. Four days after being admitted in the rehabilitation area, a baseline assessment was performed to evaluate their degree of functionality (disability and mobility) and their perception of health-related quality of life. Eight weeks later, the same assessment was carried out for monitoring purposes and to analyze the possible differences between the groups according to stroke type and with respect to the normative population data. During those weeks, they were at a rehabilitation area at a hospital.

The data in the Basque health survey referring to the application of the SF-36 questionnaire [14] were used to compare the participants' perception of their health-related quality of life with the normative scores in their geographical area of residence.

### 2.4. Material

#### 2.4.1. Tests

*(1) World Health Organization—Disability Assessment Schedule II (WHO-DAS-II) [15]*. This questionnaire assesses disability through 6 domains and a total of 36 items. Each domain consists of 6 items on a Likert scale from 1 “none” to 5 “extreme/I cannot do it.” It evaluates understanding and communicating (cognition); getting around (mobility); dressing, eating, and being alone (self-care); getting along with people/social interaction (getting along); difficulty in life activities (housework and work); and how other people and the environment make it difficult for them to participate in society (social participation). Scores range from 0 (no disability) to 100 (total disability).

The “housework” and “work” domains had very high internal consistency (*α* = .96 and *α* = .97, respectively), the “cognition,” “mobility,” and “social participation” domains had high internal consistency (*α* = .81, *α* = .88, and *α* = .85, respectively), while the “getting along” domain showed medium-high internal consistency *α* = .77 and the domain “self-care” had *α* = .71 [15].

*(2) The Timed “Up and Go” Test (TUG) [16]*. The TUG measures the individual's mobility to see if they can walk independently. Support devices may be used by people who need them. In this, the patient needs to be seated on a chair with back and arm supports. Then, the individual is asked to stand up from the chair, walk a distance of 3 meters, turn around, and sit down again adopting the original position. The patient is asked to do this once to assess their mobility and then to repeat this task 3 times. The three trials are timed (seconds), and the average time is calculated. If the person has an average of less than 10 seconds, they are considered to be “independently mobile”; if their average is less than 20 seconds, they are deemed to be “mostly independent”; people with an average between 20-29 seconds are deemed to have “variable mobility,” and those with an average higher than 29 seconds are considered to have “reduced mobility.” The test-retest and interjudge reliability was .99 [17].

*(3) SF-36 V2 Health Questionnaire [18, 19]*. This is one of the most used questionnaires for the evaluation of health-related quality of life in both the normal and clinical population. It consists of 36 items that assess health-related positive and negative aspects. These items cover 8 domains of mental and physical health. The domains are Physical Functioning (PF), Role-Physical (RP), Bodily Pain (BP), General Health (GH), Vitality (VT), Social Functioning (SF), Role—Emotional (RE), and Mental Health (MH). It also allows two score summaries to be obtained: the Physical Component Summary (PCS) and the Mental Component Summary (MCS). The scores range from 0 (worse health status) to 100 (best health status). All domains have a reliability greater than .75, and even greater (“physical functioning,” “role—physical,” and “role—emotional” have an internal consistency > .90), except for the “social functioning” domain (*α* = .74) [20].

### 2.5. Statistical Analysis

The mean (M) and standard deviation (SD) were used to describe the data in the case of interval or ratio variables, and the frequency and percentage were used for nominal variables. For making intergroup and intragroup comparisons, exact tests were used by applying SPSS-V22 [21] and/or nonparametric tests (Mann–Whitney *U*-test and Wilcoxon test). In addition, the *t*-tests and effect size estimates (Cohen's *d*) were used to compare people with a stroke (*n* = 30) with a normative group (*n* = 7410) in the perception of health-related quality of life variable (SF-36).

## 3. Results

### 3.1. Level of Functionality

#### 3.1.1. Disability

Table 2 shows the data referring to the degree of disability measured by the WHO-DAS-II, according to stroke typology for the baseline time and for the eight-week follow-up, as well as any changes that occurred between these two points in time. Regarding the differences in the degree of disability at baseline time, only a statistically marginal difference (*p* = .058) was found in the “getting along” domain, where the hemorrhagic stroke group had higher scores (*M* = 5.6) than the ischemic group (*M* = 1.2). It should be noted that these are low scores, since the range of scores of the questionnaire ranged from 0 to 100. At the 8-week follow-up, no statistically significant differences were found in any of the domains explored. When the contrast was measured at baseline time and at the 8-week follow-up, statistically significant differences were observed in all the domains except in the “getting along” domain (*p* = .150) and in the “housework” domain (*p* = .260).

#### 3.1.2. Mobility

No statistically significant data were observed either at baseline or at follow-up regarding mobility as assessed by the TUG (Table 2). However, there was a statistically significant decrease (*p* < .001) in the TUG score (*M* = 16.6 vs. 25.3 at baseline) when the change was measured after eight weeks.

### 3.2. Perception of Health-Related Quality of Life

#### 3.2.1. Hemorrhagic Stroke vs. Ischemic Stroke

Table 3 shows the results referring to the perception of health-related quality of life as evaluated by the SF-36, according to stroke type at baseline time and at the 8-week follow-up, as well as the changes that occurred between these two points in time. Statistically significant differences were seen at baseline time depending on stroke type for the “vitality” domain (*p* = .018), the “mental component summary” domain (*p* = .011), and on a trend basis, the “mental health” (*p* = .064) domain, where the hemorrhagic stroke group had higher scores than the ischemic group for all of them. At eight weeks, the comparison of the domains was not statistically significant. However, there was a statistically significant change in the perception of the health-related quality of life for all domains, except that of “bodily pain” (*p* = .692), “role—emotional” (*p* = .790), and “mental component summary” (*p* = .190).

#### 3.2.2. Stroke vs. Normative Data

**Figure 2:**
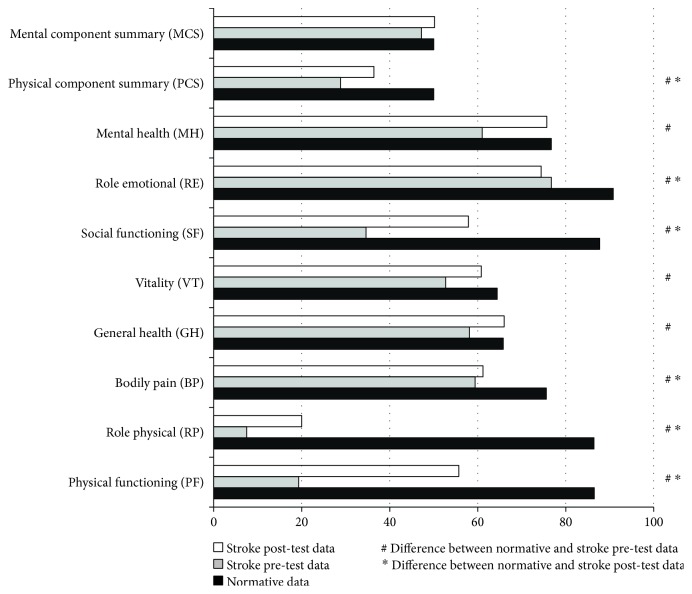
Comparison between stroke and normative data.

Table 4 and Figure 2 compare the score differences in the SF-36 between participants and the normative data from their sociodemographic area. For the baseline data (pretest), all the SF-36 domains and the “physical component summary” were statistically significant. People with a stroke had lower average scores, and the effect size was especially notable in the “physical functioning” (*d* = 3.14), “role—physical” (*d* = 2.55), “social functioning” (*d* = 2.63), and the “physical component summary” (*d* = 2.12). When considering the 8-week follow-up, the mean scores of the stroke group were close to the normative scores. The domains “general health” (*p* = .956), “vitality” (*p* = .311), and “mental health” (*p* = .755) and the “mental component summary” (*p* = .913) were similar. Nevertheless, some important differences were still found, particularly in the domains “physical functioning” (*d* = 1.44), “role—physical” (*d* = 2.14), “social functioning” (*d* = 1.47), and “physical component summary” (*d* = 1.36).

## 4. Discussion and Conclusion

The objective of this study was to compare the possible differences that exist with respect to the level of functionality and perception of health-related quality of life, depending on the type of stroke suffered, evaluated at two different points in time, and to compare the data on the perception of health-related quality of life obtained for the stroke group with the data obtained for the normative group. These factors have been analyzed by [2, 7–10]. While no evidence has been provided of a comparison of these factors between both stroke types, there is evidence of differences between risk factors, biomarkers, and mortality between both types [11–13]. Thirty patients participated in this study, of whom 70% had suffered an ischemic stroke; this prevalence of distribution by type of stroke was comparable to that found in the literature [2].

Regarding the degree of disability at the baseline, it was observed that for most domains, the hemorrhagic stroke group obtained higher scores than the ischemic stroke group, which therefore reflects that the former had a greater degree of disability. However, eight weeks later both groups had improved, while the differences between them regarding the degree of disability had decreased. The hemorrhagic stroke group improved more than the ischemic stroke group, although these differences were not statistically significant. When both groups were compared over time, there were trend differences in all domains except for the domains of “getting along” and “housework.”

Regarding mobility, no differences were observed at baseline between the groups, nor were there intergroup differences at the 8-week follow-up. However, there were statistically significant intragroup differences; that is, both groups improved their mobility.

Regarding the health-related quality of life, at baseline it was seen that the hemorrhagic stroke group scored higher than the ischemic group did and, therefore, the perception of health-related quality of life was somewhat better in this first group. However, if the mean scores of both groups were compared at baseline to the normative scores of a general population group from the same sociocultural context [22], they were well below not only the average scores of the normative population, but below a standard deviation of these scores in all SF-36 domains (Table 4). Even at the 8-week follow-up, only three domains (“general health,” “vitality,” and “mental health”) were similar to the normative scores, while the rest of the areas explored continued to show scores that indicated a reduced health-related quality of life. The comparison of the data in this study with those of the normative population [22, 23] is striking; for example, in the case of role limitations due to physical problems (physical functioning), baseline scores were much lower even for patients with hip osteoarthrosis (19.3 vs. 24.4 and 7.5 vs. 19.0, respectively). These results were representative of the significant deterioration that can occur in people affected by a stroke.

At the 8-week follow-up, the scores from both groups tended to become similar. While the members of the ischemic stroke group had improved their perception of health-related quality of life, those in the hemorrhagic stroke group had a worse perception, although these data were not statistically significant. An improvement in the perception of quality of life was observed in most of the domains of the SF-36 at eight weeks in both groups. However, the scores were still below the normative scores (except in the cases previously referred to as “general health” and “mental health”), indicating that this group had a health handicap. During the study design and recruitment of participants' process, only 30 of the 182 patients admitted could participate in the study. Noting the number of patients who could not participate in the study because of the effects of their strokes should encourage further research into this area, in order to better understand this disease and propose treatments that foster the autonomy and quality of life of these people.

Awareness of these differences means that intervention protocols and specialized rehabilitation guidelines can be developed, accelerating the patients' recovery process. The data obtained in this study indicated that patients admitted after suffering a hemorrhagic stroke should receive treatment aimed at reducing their degree of disability, while for patients who have suffered an ischemic stroke the priorities should be psychological treatment to gain a better perception on health-related quality of life and walking exercises to improve mobility. However, due to the small size of the sample, it is necessary to take these indications with caution and more studies are necessary.

In future studies, it would be appropriate to increase the number of participants to obtain better statistical power. In addition, it would also be opportune the chance to balance the comparison groups by typologies, as well as by gender.

In conclusion, the study has yielded an understanding of functionality levels and the perception of health-related quality of life referred to two types of stroke, where both had similar profiles, and their health was highly compromised with respect to the normative population. Although both groups showed an improvement in their functionality and quality of life over time, after two months they still presented scores below the norm, which indicates that substantial improvement remains to be done.

## Figures and Tables

**Table 1:** Sociodemographic characteristics of the sample.

	Total group (*n* = 30)		Ischemic group (*n* = 21)		Hemorrhagic group (*n* = 9)		Statistics			
	*n*	%	*n*	%	*n*	%	**χ** ^2^	df	*p*	*p* ^∗^
Sex										
Men	20	66.7	15	50.0	5	16.7	0.714	1	.398	.431
Women	10	33.3	6	20.0	4	13.3				
Current marital status										
Married	15	50.0	11	36.7	4	13.3	0.476	3	.924	.394
Single	10	33.3	7	23.3	3	10.0				
Widowed	2	6.7	1	3.3	1	3.3				
Cohabiting	3	10.0	2	6.7	1	3.3				
Education										
Less than primary	1	3.3	1	3.3	0	0.0	1.439	4	.837	.348
Primary	13	43.3	9	30.0	4	13.3				
High school	7	23.3	5	16.7	2	6.7				
A levels	5	16.7	4	13.3	1	3.3				
Graduate studies or Master/PhD	4	13.3	2	6.7	2	6.7				
Where they live										
Own home	24	80.0	18	60.0	6	20.0	1.429	1	.232	.329
Relative's home	6	20.0	3	10.0	3	10.0				
Who they live with										
Alone	7	23.3	6	20.0	1	3.3	1.074	1	.300	.393
With others	23	76.7	15	50.0	8	26.7				

	M	SD	M	SD	M	SD	*t*	df	*p*	*d*
Age	65.00	15.00	67.90	11.74	58.22	20.03	1.664	28	.107	0.66

Note: *χ*^2^: chi-square; df: degree of freedom; *p*: *p* value; *p*^∗^: exact *p* value; M: mean; SD: standard deviation; *t:t-*Student; *d*: Cohen's *d*.

**Table 2:** Differences between stroke typology at the two times of evaluation, and comparison of the changes over time in WHO-DAS-II and TUG.

WHO-DAS-II	Intergroup comparison												Intragroup comparison						
	*T*_0_—baseline						*T*_1_—eight-week follow-up						(all participants)						
	Hemorrhagic		Ischemic				Hemorrhagic		Ischemic				Baseline		Eight weeks				
	M	SD	M	SD	*p*	*d*	M	SD	M	SD	*p*	*d*	M	SD	M	SD	*p*	*r*	*d*
Cognition	12.8	23.1	5.00	6.3	.156	0.58	7.8	13.7	2.4	3.4	.096	0.69	7.3	13.7	4.0	8.1	.180	.362	0.25
Mobility	64.6	32.2	70.8	35.6	.656	0.18	21.5	19.8	33.6	27.4	.243	0.48	69.0	34.2	30.0	25.7	<.001	.430	1.19
Self-care	44.4	17.4	47.6	31.0	.774	0.12	21.1	16.9	19.0	21.0	.793	0.10	46.7	27.3	19.7	19.6	<.001	.333	0.97
Getting along	5.6	8.3	1.2	4.0	.058	0.79	0.9	2.8	0.8	3.6	.941	0.03	2.5	5.8	0.8	3.4	.150	.134	0.27
Housework	68.9	42.8	51.6	48.1	.360	0.37	71.1	42.3	68.4	44.0	.877	0.06	57.1	46.4	69.3	42.7	.260	.143	0.21
Soc. par	49.1	16.8	53.6	15.1	.475	0.29	43.5	7.2	43.4	18.6	.988	0.01	52.2	15.5	43.5	15.9	.010	.392	0.50
Total	39.8	12.6	37.8	13.3	.704	0.15	26.9	10.0	27.5	13.6	.906	0.05	38.5	12.9	27.3	12.4	<.001	.542	0.92
*TUG*	22.6	9.2	26.5	11.2	.267	0.36	15.9	6.8	16.9	8.2	.751	0.13	25.3	10.6	16.6	7.7	<.001	.672	1.10

Note. Soc. par: social participation. The “Work” domain was eliminated because none of the participants were working at the time of the evaluation; M: mean; SD: standard deviation; *p*: probability; *r*: Pearson's *r* correlation; *d*: Cohen's *d*.

**Table 3:** Differences between stroke typology at the two evaluation times, and comparison of changes over time in SF-36.

SF-36	Intergroup comparison												Intragroup comparison						
	*T*_0_—baseline						*T*_1_—eight-week follow-up						(All participants)						
	Hemorrhagic		Ischemic				Hemorrhagic		Ischemic				Baseline		Eight weeks				
	M	SD	M	SD	*p*	*d*	M	SD	M	SD	*p*	*d*	M	SD	M	SD	*p*	*r*	*d*
PF	20.6	18.6	18.8	26.2	.854	0.07	59.4	22.0	54.1	22.5	.557	0.24	19.3	23.8	55.7	22.1	<.001	-.120	1.06
RP	2.8	8.3	9.5	20.1	.346	0.38	27.8	36.3	16.7	29.9	.389	0.35	7.5	17.5	20.0	31.8	.080	-.085	0.33
BP	69.2	26.6	55.1	37.4	.316	0.41	68.9	33.5	57.9	35.4	.435	0.31	59.4	34.7	61.2	34.6	.692	.625	0.06
GH	61.6	13.5	56.6	19.3	.487	0.28	71.3	16.8	63.7	21.4	.353	0.38	58.1	17.7	66.0	20.2	.060	.303	0.35
V	67.2	14.4	46.4	22.9	.018	0.99	60.6	22.1	60.9	24.1	.975	0.01	52.7	22.7	60.8	23.1	.070	.452	0.34
SF	37.5	31.2	33.3	41.5	.788	0.11	65.3	32.3	54.8	38.2	.478	0.29	34.6	38.2	57.9	36.3	<.001	.449	0.59
RE	96.3	11.1	68.2	44.1	.072	0.74	81.5	37.7	71.4	46.3	.569	0.23	76.7	39.3	74.4	43.5	.790	.379	0.05
MH	71.1	20.3	56.6	18.3	.064	0.76	81.8	13.6	73.1	23.1	.303	0.42	61.0	19.7	75.7	20.8	<.001	.373	0.65
PCS	27.7	4.0	29.3	9.25	.624	0.20	38.3	8.7	35.6	7.6	.400	0.34	28.8	8.0	36.4	7.9	<.001	.114	0.72
MCS	54.7	8.46	44.0	10.3	.011	1.10	52.6	13.7	49.2	13.1	.525	0.26	47.2	10.9	50.2	13.1	.190	.498	0.25

Note. PF: physical functioning; RP: role—physical; BP: bodily pain; GH: general health; vitality; SF: social functioning, RE: role—emotional; MH: mental health; PCS: physical component summary; MCS: mental component summary; M: media; SD: standard deviation; *p*: probability; *r*: Pearson's *r* correlation; *d*: Cohen's *d*.

**Table 4:** Comparison between stroke group and normative group.

SF-36	Normative (*n* = 7410)		Stroke pretest (*n* = 30)		Stroke posttest (*n* = 30)		*t*-test comparison					
							Normative vs. pretest			Normative vs. posttest		
	M	SD	M	SD	M	SD	*t*	*p*	*d*	*t*	*p*	*d*
Physical functioning	86.5	21.4	19.3	23.8	55.7	22.1	17,15	<.001	3.14	7.86	<.001	1.44
Role—physical	86.4	31.0	7.5	17.5	20.0	31.8	13.93	<.001	2.55	11.70	<.001	2.14
Body pain	75.6	25.7	59.4	34.7	61.2	34.6	3.44	<.001	0.63	3.05	<.001	0.56
General health	65.8	19.8	58.1	17.7	66.0	20.2	2.12	.033	0.39	-0.05	.956	0.01
Vitality	64.4	19.4	52.7	22.7	60.8	23.1	3.29	.001	0.60	1.01	.311	0.18
Social functioning	87.7	20.1	34.6	38.2	57.9	36.3	14.36	<.001	2.63	8.06	<.001	1.47
Role emotional	90.8	26.1	76.7	39.3	74.4	43.5	2.94	.003	0.54	3.42	<.001	0.63
Mental health	76.7	17.5	61.0	19.7	75.7	20.8	4.90	<.001	0.89	0.31	.755	0.06
PCS	50.0	10.0	28.8	8.0	36.4	7.9	11.59	<.001	2.12	7.43	<.001	1.36
MCS	50.0	10.0	47.2	10.9	50.2	13.1	1.53	.126	0.28	-0.10	.913	0.02

Note. PCS: physical component summary; MCS: mental component summary; M: mean; SD: standard deviation; *p*: probability; *t*: *t*-Student; *d*: Cohen's *d*.

## Data Availability

The data used to support the findings of this study are available from the corresponding author upon request.
